# A stabilizing factor?–Video gaming among elite athletes during the first lockdown of the COVID-19 pandemic

**DOI:** 10.3389/fpsyg.2022.880313

**Published:** 2022-11-28

**Authors:** Tabea Breckwoldt, Stefan Fröhlich, Samuel Iff, Raoul Bitar, Jörg Spörri, Johannes Scherr, Erich Seifritz, Boris B. Quednow, Malte Christian Claussen

**Affiliations:** ^1^Department of Psychiatry, Psychotherapy and Psychosomatics, Psychiatric University Hospital Zurich, University of Zurich, Zurich, Switzerland; ^2^Sports Medical Research Group, Department of Orthopaedics, Balgrist University Hospital, University of Zurich, Zurich, Switzerland; ^3^University Center for Prevention and Sports Medicine, Department of Orthopaedics, Balgrist University Hospital, University of Zurich, Zurich, Switzerland; ^4^Experimental and Clinical Pharmacopsychology, Department of Psychiatry, Psychotherapy and Psychosomatics, Psychiatric University Hospital Zurich, University of Zurich, Zurich, Switzerland; ^5^Private Clinic Wyss AG, Muenchenbuchsee, Switzerland; ^6^Adult Psychiatry, Psychiatric Services Grisons, Chur, Switzerland

**Keywords:** video gaming behavior, mental health, COVID-19 pandemic, elite athletes, sports psychiatry, sports medicine

## Abstract

**Objectives:**

Little is known about the extent of video gaming among elite athletes, specifically under stressful conditions like those induced by the current COVID-19 pandemic. The aim of this study was to evaluate the intensity and extent of video gaming in the context of the COVID-19 pandemic, during which the usual daily routine of many athletes was disrupted.

**Methods:**

Overall, 203 elite athletes from Switzerland who participated in Olympic sports or in “International Olympic Committee”—approved disciplines were interviewed using an online questionnaire. They were questioned on their video game consumption during the first Swiss lockdown during the COVID-19 pandemic as well as on their athletic performance and economic circumstances. Additionally, mental and physical health were assessed by standardized questionnaires. From this questionnaire data, predictors of gaming time were evaluated using multivariable analysis.

**Results:**

Before the lockdown, 21% of the participating athletes played video games regularly. The average playing time was 15.8 h per month within the gamer group. During the first lockdown, 29% of athletes reported gaming regularly, and within the gamer group the average gaming time increased significantly, by 164%. The mental health burden showed significant differences between gamers and non-gamers regarding existential fears during the lockdown, the ability to cope with governmental measures due to COVID-19 and total sleeping time. However, there was no statistical difference in respect to standardized scales for depressive symptoms, sleep behavior, and anxiety. Higher video gaming time during the lockdown was significantly associated with male gender and previous gaming before the COVID-19 lockdown.

**Conclusion:**

Video gaming time increased significantly during the first lockdown. Whether video gaming among elite athletes hereby functions as an effective coping behavior remains to be shown and requires more research.

## 1. Introduction

Video gaming is a pleasant and social form of entertainment, since more than half of all video gamers cite social factors as a reason for playing (Steadman, 2019; ESA, 2021). In the U.S. it is estimated that 74% of all households have at least one member who is a video game player (ESA, 2021). Video games can be used to socialize as well as provide mental stimulation.Video games can also function as a coping strategy to escape daily life and relieve stress (Steadman, 2019; ESA, 2021). On the other hand, like many behaviors, video gaming carries a certain risk of becoming addictive, although this only affects a small proportion of gamers (Griffiths, 2005, 2010; Ballabio et al., 2017).

Despite extensive discussion of the topic, few researchers have addressed the question of gaming behavior in elite athletes, and little is known about the extent of gaming among athletes or about their levels of possibly problematic gaming behavior. This lack of objective data was also noted by the International Olympic Committee (IOC) in 2019 (Reardon et al., 2019).

As a population, elite athletes are under high pressure due to intense training plans and the demand for competitive athletic performance. They rely on their physical achievements, mental strength, and discipline (Daumiller et al., 2021), but they are also at risk of mental health problems, substance use, and addictive behavior disorders, such as gambling problems (Håkansson et al., 2018; Reardon et al., 2019).

This high-pressure routine of many athletes was drastically altered by the recent pandemic. Coronavirus disease 2019 (COVID-19) is an infectious disease caused by the “severe acute respiratory syndrome coronavirus 2” (SARS-CoV-2), and its outbreak at the beginning of 2020 led to worldwide restrictions of public life and freedom of movement. In Switzerland, on 16 March 2020, the Federal Council announced an “extraordinary situation” and restricted public life and events (BAG, 2020). As a result, many sport competitions were canceled or were undertaken without spectators, giving rise to uncertainty among athletes and sport clubs. Due to the COVID-19 pandemic, many typical elements of competitive sports were eliminated, potentially affecting competitive athletes on a motivational, emotional and performance level. It is plausible to assume that this situation brings enormous stress to the general population as well as to elite athletes, especially if their existence and professional prospects depend on performing (Claussen et al., 2020; de Quervain et al., 2021).

In this study, we seek to understand more about both typical gaming habits among athletes and how those habits were altered by the COVID-19 pandemic. We hypothesize an increased gaming time during the lockdown due to isolation restrictions. Therefore, the current survey aimed at evaluating the extent of video game consumption in relation to mental and physical health factors, such as athletic performance, anxiety, and depressive symptoms. In addition, the change in video gaming behavior was analyzed in response to the restrictions during the COVID-19 pandemic, during which the usual daily structure of the athletes was considerably disturbed.

## 2. Materials and methods

### 2.1. Data collection and setting

The extraordinary situation in the context of the COVID-19 pandemic—known as the “first lockdown”—was decreed by the Swiss Federal Council lasting from 17 March to 10 May 2020. This included the prohibition of all events and the closure of many public places including sport facilities. Private training was therefore still possible, but competitions and team training were not.

In the middle of this period, a REDCap-based online survey (REDCap 9.10.0 - © 2020 Vanderbilt University) was sent to different elite athletes in Switzerland who performed in Olympic sports or IOC-approved disciplines on a national or international level. This online questionnaire was shared by sports clubs for team sports, whereas for individual sports, it was distributed by the Swiss national sports federations. So that athletes could participate in their mother tongue, questionnaires were offered in German and French. Open questions were primarily designed in German. The French translation was validated by two people proficient in both languages and differences in translation were resolved by bilateral discussion. Regarding the standardized questionnaires, validated translations for both languages were used.

The survey evaluated the 4 weeks before the lockdown as well as the first 4 weeks of the lockdown. For both periods, athletes reported the frequency of video gaming, gaming time, and parameters related to mental health. As demographic parameters, the survey queried age, gender, type of sport and time spent on educational and/or part-time occupational duties. Video game playing time was quantified by frequency (days per month [d/m]) and mean playing time (hours per month [h/m]). We also asked whether the participants were able to earn enough income from their athletic performance to make a living. Finally, we evaluated the performance in athletes (before and during the lockdown) with three different variables: the objective activity (training time hours per day [h/d]), the subjective training intensity and the subjective maximum of athletic performance (both in [%] of the respective *z*-value). Although athletic performance is usually quantified by athletic success, this was not possible in the current study due to canceled tournaments and events.

With respect to COVID-19 itself, participants were asked whether they had been infected and about their personal quarantine requirements. Furthermore, the questionnaire evaluated to what extent the athletes worried about their athletic career due to the COVID-19 pandemic and how they were personally affected by the lockdown measures.

To understand the psychological effects of the situation, depressive symptoms, sleep behavior, and anxiety characteristics were evaluated using the Patient Health Questionnaire (PHQ9), the Insomnia-Severity-Index (ISI), and certain aspects resp. questions extracted from the Pittsburgh Sleep Quality Index, as well as the Spielberger State-Trait-Anxiety-Index (STAI) (Spielberger et al., 1983; Buysse et al., 1989; Bastien et al., 2001; Kroenke et al., 2001). Also, substance use was evaluated, including alcohol and cannabis (frequency of consumption).

The aspects of fear and anxiety were evaluated in three different questions and standardized questionnaires using the STAI survey. The athletes were asked whether they experienced existential fears (4 weeks before and/or during the lockdown), whether they worried about their careers due to the pandemic, and how well they were able to cope with the restrictions established by the Swiss government. For each of these questions, participants had to choose a value on a scale from 0 to 100, where zero corresponded to “I have no existential fears,” “I have no worries at all about my career due to COVID-19,” and “I cannot cope at all with the measures due to the pandemic.” At the other extreme, the value 100 expressed “I experience very strong existential fears,” “I have major worries about my career due to COVID-19,” and “I cope very well with the measures due to the pandemic.” Additionally, all participants filled out the STAI questionnaire regarding their anxiety traits and states (Spielberger et al., 1983; Spielberger, 2010).

To gain an understanding of the physical complaints of the participants, current health problems like traumatic/overuse injuries or illnesses were assessed using the Oslo Sports Trauma Research Centre (OSTRC) questionnaire on health problems (Clarsen et al., 2013). For the classification of self-reported injuries or illness (yes/no), the first question of the OSTRC was used (“Did you have any difficulties participating in normal training and competition due to injury, illness or other health problems during the past 4 weeks?”).

### 2.2. Characteristics of the sample

Inclusion criteria were training volume (before the COVID-19 lockdown) of at least 1 h/d and a minimum age of 18. Participants with incomplete data and those who did not participate in Olympic disciplines or in sports recognized by the IOC were excluded. There was no compensation for participation in this study.

#### 2.2.1. Gamer type

Gaming time was calculated by multiplying the number of gaming days per month by the time spent gaming per day [h/d], resulting in a gaming time [h/m]. With respect to the range of gaming time within our sample, gamer types were classified as “no gamer” (<1 h/m), “occasional gamer” (between 1 h/m and <10 h/m), “moderate gamer” (≥ 10 h/m) and “frequent gamer” (>30 h/m), which is similar to the classifications used in a study from Rehbein et al. (2010). In this manner, we could characterize the behavior and observe possible trends.

#### 2.2.2. Change in gaming behavior

With respect to the change in gaming behavior before and during the first lockdown, the sample was divided into the four groups “never gamers,“same gaming time,” “less gaming time” and “more gaming time.” A change in gaming behavior was defined as a change of at least 20% compared to the pre-lockdown gaming time, which corresponds to –0.5 or 0.5 z-score change, rounded to the nearest 10%.

### 2.3. Data evaluation and measure calculations

Baseline data was expressed as mean ±SD and frequency tables for categorical data. Demographic data of the gamers and non-gamers were compared using the two-tailed independent sample *t*-test for continuous data and χ^2^ tests for categorical data. In tables with more than two groups, χ^2^ test was used for tables with more than four fields, one-way analysis of variance for continuous variables, and Kruskal-Wallis rank tests for variables that violated the assumptions of parametric tests. Differences between the groups were considered significant at *p* < 0.05. For gaming behavior during the lockdown, we fitted a general linear model (GLM) using current gaming time as the dependent variable, previous gaming as the independent variable and gender, team sports, injury, activity, pandemic measures, and occupation percentage as covariates. In an attempt to adjust for psychiatric measures, we added the scores of PHQ9, ISI, TRAIT, and STATE, as well existential fears and worries about career to the model. All explanatory variables that had an association with gaming at *P* < 0.20 in the univariable analyses were included in the multivariable-adjusted analyses. Using a stepwise backward elimination process, the least significant variables were then removed from the base model. Only variables with *P* < 0.05 remained in the final parsimonious model.

Dominance analysis was additionally performed as was the calculation of the correlation coefficients between all variables.

### 2.4. Ethical approval

The local ethics committee accepted this survey by a declaration of non-responsibility (KEK-ZH-NR: Req-2020-00408).

## 3. Results

### 3.1. Baseline parameters

In total, 203 athletes answered the survey, 193 of whom provided information on their gaming behavior: 87 women (45%) and 106 men (55%), with a mean age of 24.1 years (range 18–38 years). Within this sample, winter (*n* = 94, 49%) and summer (*n* = 99, 51%) sports were represented equally, and 59 athletes (31%) were active in team sports. Alongside their activity in sports 116 (60%) of these athletes were employed or studying, with an average workload of 67.5%. From their performance in sports alone, 101 (52%) earned sufficient income, meaning that 8% of the athletes were working or studying even though they earned enough money from their athletic performance alone. Three (2%) had a positive SARS-CoV-2 test during the first period of the lockdown (March and April 2020), and 10 athletes (5%) had to be in quarantine, with an average time of 10.8 days (SD 5.9).

### 3.2. Gaming behavior under regular circumstances

Fifty-two (27%) athletes stated “yes,” when they were asked if they played video games 4 weeks before the pandemic, and 41 (21%) athletes were considered as playing regularly (≥ 1 h/m), with an overall average playing time of 14.6 h/m (SD 17.1) distributed over 9 days per month. Maximum reported playing time was 93 h/m, averaging roughly 3 h/d. Thirty-six gamers (88%) were male and five (12%) were female. The proportion of individual athletes who gamed was 17.2%, whereas 30.5% of team athletes gamed, which represents a significantly higher proportion (*P* < 0.05). Athletes who gamed regularly tended to work or study more, reporting a higher workload. We found no statistical difference between gamers and non-gamers regarding athletic performance, substance use or self-reported injuries or illness.

Despite spending the same amount of time in bed, gamers reported significant 0.4 h less effective total sleep duration than non-gamers (*p* = 0.027). In the context of COVID-19 related issues, video gamers coped noticeably better with the measures of the government due to the pandemic (*P* < 0.05) and worried less about their career due to the COVID-19 pandemic. Overall, athletes who gamed regularly experienced less existential fear in general, and significantly less during the first lockdown (*P* < 0.05). Six (15%) of the gamers reported an injury or an illness compared to 42 (28%) athletes in the non-gamer group (also see Tables 1, 2).

**Table 1:** Characteristics of professional athletes who game regularly compared to non gaming professional athletes.

	**Gamer (*n* = 41)**	**Non gamer (*n* = 152)**	***P*-value**
Age	23.4 years (SD 5.1)	24.3 years (SD 5.2)	0.355
Gender	12% women 88% men	54% women 46% men	<0.001^***^
Sports type^a^	44% summer 56% winter	53% summer 47% winter	0.297
	44% team 56% individual	27% team 73% individual	0.055
Paid occupation^a^	63%	59%	0.720
Workload	73% (SD 23.9)	66% (SD 29.5)	0.253
Sufficient income from sport^a^	51%	53%	1.000
Substances^a^			
Alcohol	30 (73%)	93 (61%)	0.200
Days/ month	3.3 (SD 3.6)	3.2 (SD 3.2)	0.829
Cannabis	1 athlete	2 athletes	1.000
Days/ month	<1	0.8 (SD 0.5)	0.270
COVID-19 related issues^b^			
Positive test	0%	2% (*n* = 3)	1.000
Quarantine	1 athlete	9 athletes	0.692
Duration of quarantine	14 days	10.3 days (SD 6.2)	0.595
Coping with COVID-19 restrictions	82.5% (SD 14.3)	72.3% (SD 22.0)	0.005^**^
Sleep^b^			
Time in bed	8.6 (SD 0.9)	8.8 (SD 1.0)	0.155
Total sleeping time	7.6 (SD 1.0)	8.0 (SD 1.1)	0.027^*^
Self-reported injuries or illness^b^	6 (15%)	42 (28%)	0.105

Categorization “gamer” (>1 h/month) resp. “non-gamer” (<1 h/month) was assessed in respect to the period before the lockdown; *n* = 193, 41 gamers, 152 non-gamers.

a Variables concerning the time before the lockdown.

b Variables that were assessed during the lockdown.

* *p* < 0.05,

** *p* < 0.01, and

*** *p* < 0.001.

**Table 2:** Athletic performance, fears, depressive symptoms, and sleep behavior in professional athletes who gamed regularly before the lockdown.

	**Gamer**				**Non gamer**	***P*-value**
	**Total**	**Minimal (<10 h/m)**	**Moderate (≥10 h/m)**	**High (>30 h/m)**		
**Athletic performance**						
**before COVID-19** ^*a*^						
Training time [h/d]	2.9 (SD 1.0)	2.9 (SD 1.0)	2.6 (SD 1.1)	3.4 (SD 0.9)	3.2 (SD 1.5)	0.178
Training intensity [%]	72.5 (SD 20.2)	71.4 (SD 19.6)	83.4 (SD 13.9)	54.2 (SD 23.2)	74.7 (SD 19.2)	0.518
Training maximum [%]	79.7 (SD 15.4)	79.4 (SD 15.2)	84.1 (SD 13.3)	71.6 (SD 19.7)	77.9 (SD 16.4)	0.530
**Fear and Anxiety**						
Existential fears						
Before lockdown period^*a*^	11.2 (SD 19.5)	15.3 (SD 23.6)	3.0 (SD 6.4)	9.0 (SD 7.8)	15.1 (SD 21.7)	0.296
During lockdown^*b*^	14.4 (SD 24.8)	18.6 (SD 29.3)	5.5 (SD 12.9)	12.8 (SD 15.2)	24.3 (SD 26.9)	0.035*
Career worries^*b*^	27.4 (SD 26.9)	29.1 (SD 23.1)	27.1 (SD 34.4)	20.0 (SD 31.5)	36.6 (SD 28.8)	0.068
Coping with COVID-19 restrictions^*b*^	82.5 (SD 14.3)	81.9 (SD 15.4)	82.4 (SD 14.0)	86.0 (SD 10.6)	72.3 (SD 22.0)	0.005*
STAI score trait^*b*^	26.9 (SD 12.8)	30.4 (SD 12.9)	23.2 (SD 12.2)	17.0 (SD 5.8)	27.3 (SD 10.5)	0.865
STAI score state^*b*^	29.8 (SD 12.6)	33.6 (SD 12.8)	25.5 (SD 10.7)	18.8 (SD 5.0)	29.1 (SD 11.1)	0.732
**Depressive symptoms, sleep**,						
**and substance use**						
PHQ-9 score^*b*^	4.3 (SD 3.5)	4.9 (SD 3.7)	3.7 (SD 3.1)	3.0 (SD 3.0)	4.6 (SD 3.1)	0.697
ISI score^*b*^	5.0 (SD 4.6)	5.7 (SD 5.3)	4.4 (SD 3.5)	3.0 (SD 2.0)	5.6 (SD 4.0)	0.472
Total sleeping time [h/n]^*b*^	7.6 (SD 1.0)	7.5 (SD 1.0)	7.7 (SD 1.3)	7.8 (SD 0.8)	8.0 (SD 1.1)	0.027^*^
Time in bed [h/n]^*b*^	8.6 (SD 0.9)	8.5 (SD 0.9)	8.7 (SD 1.0)	8.8 (SD 1.1)	8.8 (SD 1.0)	0.155
Drinking alcohol^*a*^	27 (66%)	17	9	1	78 (51%)	0.113
Alcohol [d/m]^*a*^	6.6 (SD 6.3)	6.9 (SD 7.0)	6.6 (SD 5.3)	2.0	5.0 (SD 3.9)	0.119

*n* = 193: “minimal” (*n* = 25), “occasional” (*n* = 11), “frequent” (*n* = 5), “non-gamer” (*n* = 152). *P*-values refer to the total gamers compared to non gamers.

a Variables concerning the time before the lockdown.

b Variables that were assessed during the lockdown.

* *p* < 0.05.

### 3.3. Change of gaming behavior due to the COVID-19 pandemic

During the first period of the lockdown, 63 athletes (33%) stated “yes” to the question of whether they had played video games within the last 4 weeks, and 29% of the athletes reported playing video games regularly (>1 h/d) with a mean playing time of 21.8 h/m (SD 22.8). Playing time increased by 165% within the group of athletes who used to play video games before the isolation period.

In our sample, 41 athletes spent more time video gaming, 14 athletes less time, 18 athletes did not change their gaming time and 120 athletes never gamed, neither before nor during the first lockdown. Athletes who gamed more during the lockdown increased their gaming time from 5.5 to 21.8 h/m. and, thus spending on average 16.3 h/m more on video gaming than before. In particular, the group of “occasional” gamers increased their gaming time from 4.6 to 12.6 h/m, therefore, increased the playing time on average by 8.0 h/m [SD 14.3]. Maximum gaming time was 84 h/m.

Compared to all other athletes, those who gamed more tended to be male and used to already play video games before the lockdown (for all applies *P* < 0.05). Again, summer and winter sports were distributed evenly between the groups, and groups did not differ in their outside employment status or in earning sufficient income from sport. However, team athletes tended to spend significantly more time video gaming during the lockdown than those involved in individual sports (*P* < 0.05). While 40% (23) of the team athletes were gaming regularly, only 23% (32) of the individual athletes did so. Those who gamed “more” also reported a significantly higher workload when employed or studying (79.2% [SD 25.9]) in comparison to the groups that gamed “less” (67.8% [SD 22.8]), the “same” (74.0% [SD 28.0]) or “never” (63.5% [SD 29.1]) (*P* < 0.05). Twenty-one athletes started playing video games during the first lockdown and 10 athletes stopped playing video games.

Regarding performance levels, fears, depressive symptoms, sleep, and substance use, the groups “same,” “never,” “more,” and “less” showed no statistical difference within the context of the first lockdown (also see Table 3).

**Table 3:** Change in gaming behavior in the context of the first lockdown due to the COVID-19 pandemic.

	**Consistent gaming behavior**		**Change in gaming behavior**			
	**Never (*n* = 120)**	**Same (*n* = 18)**	**Less (*n* = 14)**	**More (*n* = 41)**	**Total**	***p*-value**
**Video gaming**						
Δ gaming time in relation						
to the previous gaming time[%]	-	99.3 (SD 5.5)	21.8 (SD 30.8)	477.1 (SD 768.2)	264.7 (SD 570.4)	0.060
Δ absolute gaming time [hours/month]	-	0.9 (SD 5.5)	-5.4 (SD 9.1)	16.3 (SD 16.2)	3.2 (SD 10.6)	<0.001^***^
**Athletic performance**						
Δ training time in relation						
to the previous activity[%]	99.1 (SD 77.1)	91.1 (SD 33.3)	85.6 (SD 38.5)	105 (SD 56.7)	98.6 (SD 67.7)	0.774
Δ training intensity [%]	–12.4 (SD 26.7)	–9.2 (SD 23.7)	–12.0 (SD 29.1)	–15.1 (SD 25.0)	–12.7 (SD 26.1)	0.874
Δ training maximum [%]	–10.0 (SD 20.4)	–9.4 (SD 17.0)	–6.5 (SD 23.5)	–11.2 (SD 24.8)	–9.9 (SD 21.2)	0.916
**Fears and anxiety**						
Existential fears						
Before isolation period	15.8 (SD 21.9)	16.8 (SD 27.1)	7.8 (SD 16.2)	11.0 (SD 17.4)	14.3 (SD 21.2)	0.374
During the lockdown	25.9 (SD 26.8)	19.2 (SD 31.5)	10.0 (SD 23.5)	16.8 (SD 23.6)	22.2 (SD 26.7)	0.066
Career worries	36.8 (SD 28.8)	33.3 (SD 35.1)	34.5 (SD 30.0)	29.1 (SD 24.5)	34.7 (SD 28.6)	0.528
Coping with COVID-19 restrictions	72.7 (SD 22.2)	76.9 (SD 19.3)	82.3 (SD 10.8)	75.9 (SD 20.5)	74.5 (SD 21.0)	0.365
STAI score trait	27.5 (SD 10.8)	23.4 (SD 10.5)	33.8 (SD 12.1)	25.8 (SD 10.8)	27.2 (SD 11.0)	0.047^*^
STAI score state	28.9 (SD 11.2)	26.1 (SD 11.1)	34.9 (SD 14.7)	29.4 (SD 10.7)	29.2 (SD 11.4)	0.190
**Depressive symptoms**,						
**Sleep and substance use**						
PHQ-9 score	4,6 (SD 3,2)	4,1 (SD 2,8)	5,9 (SD 3,6)	4,0 (SD 2,8)	4.5 (SD 3.2)	0.261
ISI score	5,3 (SD 4,1)	5,8 (SD 4,8)	8,1 (SD 4,1)	4,8 (SD 3,6)	5.4 (SD 4.1)	0.071
Total sleeping time	8.0 (SD 1.1)	7.6 (SD 0.9)	7.6 (SD 1.3)	8.0 (SD 1.2)	7.9 (SD 1.1)	0.553
Time in bed	8.8 (SD 0.9)	8.5 (SD 1.2)	8.6 (SD 0.9)	8.8 (SD 1.2)	8.8 (SD 1.0)	0.610

* *p* < 0.05 and

*** *p* < 0.001.

### 3.4. Multivariable analysis

Except for male gender and the fact that the athlete used to play before the COVID-19 lockdown, we found that video gaming time during the lockdown was not affected at a statistically significant level by any of the other variables we evaluated (type of sport, training, occupation, aspects of anxiety, and fears, depressive symptoms and disordered sleeping). In this multivariable regression of gaming time during the lockdown, the influence of male gender was significant, with *p* < 0.05, and athletes who already regularly played video games before the lockdown were very likely to also play during the lockdown (*p* < 0.01) (also see Table 4). The statistical power of the final parsimonious regression model is >0.9. Dominance analysis was additionally performed and resulted in the same model as the backwards elimination of the GLM. Results of the dominance analysis are shown in Supplementary Table S5. Due to possible unstable results of stepwise regression, we provide the correlation matrix in order to show all relations between the used variables, presented in Supplementary Table S6.

**Table 4:** Coefficients of multivariable regression of gaming time during the lockdown.

	**Univariable model**	**Adjusted multivariable model (AMM)**	**Final parsimonious (AMM)**
	**Gaming during**	**Gaming during**	**Gaming during**
	**[h/m]**	**[h/m]**	**[h/m]**
Gaming before the lockdown [h/m]	1.050^***^	0.982^***^	0.973^***^
Gender	–10.436^***^	–5.378^**^	-
			5.226^***^
Team sport	7.722^***^	3.289	3.322^*^
Training activity during the lockdown [h/d]	–0.611	0.439	
Occupation	0.371	1.175	
Fears about sports career due to COVID-19	–0.057	–0.022	
Existential fears due to COVID-19	–0.062	0.011	
Coping with COVID-19 restrictions	0.049	–0.036	
STAI score trait	–0.181	–0.044	
STAI score state	–0.144	0.011	
PHQ-9 score	–0.406	0.119	
ISI score	–0.366	–0.129	
Self-reported injury/illness	–0.026	2.447	

Multivariable regression analysis was conducted with respect to gaming time before the lockdown, gender, type of sport, training, occupation, aspects of anxiety and fears, depressive symptoms, and disordered sleeping.

* *p* < 0.05,

** *p* < 0.01, and

*** *p* < 0.001.

### 3.5. Internal and external validity

Internal validity is judged to be according to current standards and reported according to STROBE (Cuschieri, 2019). The generalisability however is only possible with professional athletes in a similar setting.

## 4. Discussion

With this evaluation, we were able to describe the gaming behavior of elite athletes on a quantitative level. Approximately 1/5 of elite athletes play video games regularly under normal circumstances, with an average time of 14.6 h/m (SD 17.1) and a frequency of 9 d/m (SD 8.6). During the first lockdown, which lasted from the 17th of March to 10th of May 2020, gaming time within the gamer group increased by 164.7%. In addition, the overall percentage of athletes consuming video games rose by 8%.

Gaming time during the lockdown was higher for males and for those athletes who had played before the lockdown, but otherwise we found no other significant influence on the time spent playing.

### 4.1. Video gaming behavior and the mental health of elite athletes before and during the COVID-19 pandemic

The gaming time of elite athletes is considerably low in the context of studies concerning gaming time and problematic gaming. Rehbein et al. found that 36% of the general adult population in Germany played video games for 44 h/m on average. Of these, 3.5% played occasionally (at least once in 12 months), 28.2% were “normal” players (daily gaming time up to 2.5 h) and 4.2% were classified as “frequent” (daily gaming time >2.5 h) (Rehbein et al., 2010). Other authors have defined “dedicated gamers” as those who played for approximately 30 h per week and “very excessive gaming” as up to 80 h per week, i.e., four times the maximum playing time within our sample (Chappell et al., 2006; Griffiths, 2010; Ballabio et al., 2017). Therefore, active elite athletes seem to spend less time video gaming, and fewer of them consume video games than among the general population.

Also, the gender gap is notable: only 12% of gamers in our sample were female, which does not follow the current trend of the general population, where the proportion of women is 45% (ESA, 2021).

To date, it has often been hypothesized that elite athletes may be prone to problem gaming (Håkansson et al., 2018; Reardon et al., 2019; Bitar et al., 2022) and it can in fact be challenging to distinguish between “enthusiastic” gaming and “problematic” gaming. There is as yet no clear differentiation between these behaviors, as this is still a clinical issue requiring more research (APA, 2013; Király et al., 2015). The existing orienting criteria for defining problematic gaming are dichotomous, and defined by the circumstance, that the patient is severely impaired in his performance or functionality over a time period of at least 12 months (APA, 2013). It is therefore not likely to find this level of gaming behavior among functioning elite athletes. Also, in this survey, no standardized screening tool for problematic gaming was used, so we are not able to make definite statements regarding problematic gaming. However, in our sample, we detected no excessive gaming time, severe performance impairment, or higher level of mental symptoms within the gamer group.

The situation after the outbreak of the COVID-19 pandemic can be described as an unexpected and significant disruption of the athletes' lives and perspectives. Such situations require behavioral adaptations and high cognitive and emotional flexibility (Pété et al., 2022). Also, several studies showed reduced well-being, sleep quality and physical performance in team athletes during isolation period or during COVID-19 infection, arguing that awareness of the effects of COVID-19 on elite team athletes need to be established (Mon-López et al., 2020; Wagemans et al., 2021).

As expected, during the first weeks of the first lockdown, gaming time increased significantly, especially for those athletes who had only occasionally gamed before. In this particular situation, video gaming appears to be an effective part of the adaptation process; with the restrictions on movement and limitation of training possibilities, athletes could interact with friends and team colleagues through gaming. The proportion of team athletes in the sample population who increased their playing time was significantly higher than in individual sports. This may be due to the more drastic restrictions in team training and fewer limitations on individual training: team athletes had more spare time and may have been missing the social component of team training. This thesis corresponds to a study from Italy, in which athletes reported that being in contact with colleagues and coaches through the internet was helpful when managing the lockdown (di Fronso et al., 2022).

Athletes who had regularly gamed before the pandemic tended to be less anxious, had fewer depressive symptoms, and displayed lower scores regarding sleep disturbances. However, no statistical significance could be found. We did not find significant impairments in athletic performance before the pandemic and in the first period of the lockdown. Also, among athletes with a secondary employment or those who were studying alongside their sports, gamers tended to have a higher workload than non-gamers. Athletes who gamed regularly seemed to cope significantly better with the measures of the Swiss government.

Therefore, video games may have been used as a positive coping mechanism, similar to seeking social support and to active stress coping. Elite athletes are used to deal with stressors due to their intense training and competition routine (Daumiller et al., 2021). Hence, it is likely they were able to use video gaming as a coping strategy under the circumstances of the first lockdown.

### 4.2. Limitations

As a key limitation of this study, the absence of a control group must be mentioned. The questionnaires were voluntary and based on self-reporting. Therefore, they may suffer from certain biases, such as selective recall due to impaired memory recall and social desirability. Also, the recruited sample of professional athletes in Switzerland may not be representative due to selection bias. Consequently, external validity could be impaired. Furthermore, the differences between the gamer types must be interpreted with caution due to small sample sizes, especially in the group of “frequent gamers.” Another bias, that should not be neglected, is the constellation of the “gamers,” consisting primarily of male team-athletes. Hence, any observed tendency of the “gamers” could also originate from this factor. Another note of caution, when interpreting the results, is the fact, that stepwise regression can throw out unstable results. Finally, it has to be kept in mind that athletes' careers tend to start at an early age and often last less than two decades. In our analysis we evaluated a sample of young and active elite athletes. We cannot estimate the video gaming behavior after the end of a sports career or after a major injury or illness. Also, more qualitative, contextual information would be desirable, e.g., motives and game types. Thus, further follow-up data is needed to evaluate the stability of our results with respect to the psychological symptoms.

### 4.3. Perspective

Our findings suggest that playing video games may have a positive functional role for active elite athletes. Especially in the context of the COVID-19 pandemic, it appears to help with an effective adaptation to the current circumstances. Pété et al. (2022) evaluated different coping profiles in different athletes during the COVID-19 pandemic in France, pointing out the importance of finding mechanisms to cope with the impact of pandemic on the mental health of elite athletes on a long-term basis. In this regard, video games could open up many helpful possibilities for sports clubs and athletes in terms of athletic training and social interaction. Since neurobiological studies have proven that some types of video games are beneficial to neuroplasticity (e.g., strategy, 3D adventure, puzzles), athletes could also continue training their fine motor skills and reaction time in order to maintain at least some of their athletic requirements (Brilliant T et al., 2019). In respect to further research and transfer of our data, prospective studies with unexpected and sudden events, that are defined by less training, less social interaction, change in social framework and the loss of planned successes, could be promising in order to elaborate video gaming as a coping mechanism. For example, injuries, non-consideration, or end of career show these characteristics for elite athletes.

Ultimately, video gaming may act as a stabilizing factor for elite athletes in the context of the COVID-19 pandemic and similar stressful situations.

## Data availability statement

The raw data supporting the conclusions of this article will be made available by the authors, without undue reservation.

## Ethics statement

The studies involving human participants were reviewed and approved by the Local Ethics Committee of the City Zurich by a declaration of non-responsibility (KEK-ZH-NR: Req-2020-00408). The patients/participants provided their written informed consent to participate in this study.

## Author contributions

MC, SF, JSp, and JSc conceptualized and designed the study. SF and MC recruited the participants and collected the data. SI and TB processed the data and performed the statistical analysis. TB, SI, MC, and BQ interpreted the results. TB drafted the first version of the manuscript, under supervision of MC, BQ, and SI. TB drafted the submitted manuscript, as well as the revision. All authors substantially contributed to the interpretation of data and revised them critically, approved the final version of the manuscript, and agreed to be accountable for all aspects of the work.

## Conflict of interest

The authors declare that the research was conducted in the absence of any commercial or financial relationships that could be construed as a potential conflict of interest.

## Publisher's note

All claims expressed in this article are solely those of the authors and do not necessarily represent those of their affiliated organizations, or those of the publisher, the editors and the reviewers. Any product that may be evaluated in this article, or claim that may be made by its manufacturer, is not guaranteed or endorsed by the publisher.
