# Supplementary material for: A stabilizing factor?–Video gaming among elite athletes during the first lockdown of the COVID-19 pandemic
Source: Front Psychol. 2022 Nov 28;13:880313. doi: 10.3389/fpsyg.2022.880313 (PMC9742546; doi:10.3389/fpsyg.2022.880313)
Supplement: Supplementary file 1 [file Data_Sheet_1.pdf]

SUPPLEMENTARY MATERIAL

Table 1. Dominance analysis

Gaming time during the lockdown [h/m]	Dominance Stat.	Standardized Dominized Stat.	Ranking
Gaming time before the lockdown [h/m]	0.4232	0.7716	1
Gender	0.0690	0.1258	2
Team sport	0.0284	0.0517	3
Training activity during the lockdown [h/m]	0.0017	0.0031	11
Occupation	0.0007	0.0012	13
Fears about sports career due to COVID-19	0.0046	0.0084	5
Existential Fears	0.0028	0.0051	9
Coping with COVID-19 restrictions	0.0016	0.0029	12
STAI score trait	0.0053	0.0097	4
STAI score state	0.0028	0.0052	8
PHQ-9 score	0.0020	0.0036	10
ISI score	0.0035	0.0063	6
Self-reported injury/ illness	0.0030	0.0055	7

Dominance analysis was performed and resulted in the same model as the backwards elimination of the GLM.

Table 2. Correlation coefficients

Variables	(1)	(2)	(3)	(4)	(5)	(6)	(7)	(8)	(9)	(10)	(11)	(12)	(13)	(14)
(1) Gaming time during the lockdown [h/m]	1.0													
(2) Gaming time before the lockdown [h/m]	0.668***	1.0												
(3) Gender	-0.255***	-0.218***	1.0											
(4) Sport type/ Team sport	0.032	0.058	-0.126**	1.0										
(5) Self-reported injury/ illness	-0.026	-0.051	-0.012	0.019	1.0									
(6) ISI score	0.09	0.066	-0.025	-0.065	0.1*	1.0								
(7) PHQ-9 score Existential Fears	0.133**	0.059	-0.013	-0.109*	0.137**	0.617***	1.0							
(8) STAI?	0.007	-0.019	0.029	-0.044	0.131**	0.516***	0.692***	1.0						
(9) STAI ?	-0.009	-0.023	0.037	-0.049	0.194***	0.468***	0.677***	0.792***	1.0					
(10) Existential fears due to COVID-19	-0.028	-0.004	0.048	-0.134**	0.062	0.152***	0.258***	0.245***	0.297***	1.0				
(11) Training activity during the lockdown [h/m]	-0.196***	-0.123**	0.086	-0.071	-0.014	-0.176***	-0.304***	-0.228***	-0.178***	0.239***	1.0			
(12) Coping with COVID-19 restrictions	0.002	0.046	0.039	0.123**	-0.203***	-0.211	-0.475***	-0.275***	-0.294***	-0.268***	0.109*	1.0		
(13) Occupation	0.02	0.003	-0.009	0.038	0.024	0.085	0.125**	0.08	0.048	-0.280***	-0.351***	-0.027	1.0	
(14) Fears about sports career due to COVID-19	0.042	-0.053	-0.039	-0.101*	0.076	0.227***	0.308***	0.247***	0.247***	0.210***	-0.037	-373***	-0.023	1.0

Correlation matrix of the variables used in the stepwise regression model.*p<0.05, **p<0.01, ***p<0.001.
